# The “Buruli Score”: Development of a Multivariable Prediction Model for Diagnosis of *Mycobacterium ulcerans* Infection in Individuals with Ulcerative Skin Lesions, Akonolinga, Cameroon

**DOI:** 10.1371/journal.pntd.0004593

**Published:** 2016-04-05

**Authors:** Yolanda K. Mueller, Mathieu Bastard, Patrick Nkemenang, Eric Comte, Geneviève Ehounou, Sara Eyangoh, Barbara Rusch, Earnest Njih Tabah, Laurence Toutous Trellu, Jean-Francois Etard

**Affiliations:** 1 Epicentre, Paris, France; 2 Médecins Sans Frontières, Geneva, Switzerland; 3 Centre Pasteur Cameroon, Yaounde, Cameroon; 4 National Yaws, Leishmaniasis, Leprosy and Buruli Ulcer Control Programme, Ministry of Public Health, Yaounde, Cameroon; 5 Geneva University Hospitals, Geneva, Switzerland; 6 Institut de Recherche pour le Développement (IRD) UMI 233 – INSERM U 1175 – Montpellier University, Montpellier, France; Fondation Raoul Follereau, FRANCE

## Abstract

**Background:**

Access to laboratory diagnosis can be a challenge for individuals suspected of Buruli Ulcer (BU). Our objective was to develop a clinical score to assist clinicians working in resource-limited settings for BU diagnosis.

**Methododology/Principal Findings:**

Between 2011 and 2013, individuals presenting at Akonolinga District Hospital, Cameroon, were enrolled consecutively. Clinical data were collected prospectively. Based on a latent class model using laboratory test results (ZN, PCR, culture), patients were categorized into high, or low BU likelihood. Variables associated with a high BU likelihood in a multivariate logistic model were included in the Buruli score. Score cut-offs were chosen based on calculated predictive values. Of 325 patients with an ulcerative lesion, 51 (15.7%) had a high BU likelihood. The variables identified for the Buruli score were: characteristic smell (+3 points), yellow color (+2), female gender (+2), undermining (+1), green color (+1), lesion hyposensitivity (+1), pain at rest (-1), size >5cm (-1), locoregional adenopathy (-2), age above 20 up to 40 years (-3), or above 40 (-5). This score had AUC of 0.86 (95%CI 0.82–0.89), indicating good discrimination between infected and non-infected individuals. The cut-off to reasonably exclude BU was set at scores <0 (NPV 96.5%; 95%CI 93.0–98.6). The treatment threshold was set at a cut-off ≥4 (PPV 69.0%; 95%CI 49.2–84.7). Patients with intermediate BU probability needed to be tested by PCR.

**Conclusions/Significance:**

We developed a decisional algorithm based on a clinical score assessing BU probability. The Buruli score still requires further validation before it can be recommended for wide use.

## Author Summary

In most Buruli ulcer (BU) endemic areas, laboratory diagnosis is hard to access and comes at a high cost. Clinicians are in need of new tools to assist them in identifying which patients truly require additional work-up and which can be treated directly. We analyzed the clinical data of all patients with ulcerative skin lesions that presented to Akonolinga District Hospital in Cameroon and identified which parameters were associated with BU diagnosis. We attributed a certain number of points to each parameter to build a “Buruli score”. Based on score results, clinicians can be advised either to directly treat BU (score ≥4), to look for another diagnosis (score <0) or to do a PCR test (score between 0 and 3). This algorithm was found to have a good performance. Only one out of four patients still needed an additional laboratory test to be classified between BU and non-BU. However, this score still requires validation in another context before it can be recommended elsewhere.

## Introduction

Buruli ulcer (BU) is a skin infection due to *Mycobaterium ulcerans*. Classical cases with painless ulcerated plaques and undermined edges are believed to be relatively easy to diagnose clinically in endemic regions [1]. Nevertheless, without laboratory testing to confirm the findings, diagnostic errors are probably highly under-estimated. The most common laboratory technique is the direct examination by microscopy of dry skin swabs stained with Ziehl-Neelsen (ZN) in search of alcohol-resistant bacilli [2]. PCR for insertion sequence IS2404 is very specific and currently the most sensitive diagnostic method [3]. However, it is rarely available on site and samples need to be sent to a reference laboratory and results be sent back to the clinician, resulting in substantial treatment delays and loss-to-follow-up. Cost of the method can be a barrier for the patient outside of projects supported by NGOs or research institutions.

Because of the challenges of PCR-based diagnosis of BU, alternative methods should be explored. While rapid point-of-care tests are currently being developed [4], identifying which patients would truly benefit from a diagnostic test can reduce patient expenses and treatment delays for patients with a high probability of BU. Diagnostic scores derived from multivariable prediction models can be useful in clinical decision-making [5]. These scores are based on diagnostic performance of various characteristics of medical history and clinical examination. For BU diagnosis, it has been proposed to combine ZN microscopy and PCR, only performing PCR in ZN-negative specimen, in order to reduce costs and keep diagnosis available at peripheral level for many patients [6]. Therefore, we aimed to identify clinical predictors of BU diagnosis, in order to develop a multivariable prediction model (“Buruli score”), to assist clinicians working in resource-limited settings.

## Methods

Between 2011 and 2013, a prospective cohort study was conducted in Akonolinga Health District, central Cameroon, one of the endemic areas for BU in the country [7]. All individuals presenting at Akonolinga District Hospital with a skin lesion suspect of new BU (defined as a nodule, a plaque, localized swelling and/or an ulcer in an individual residing in or having spent at least one night in a known *M*. *ulcerans* endemic area, without an obvious cause to the lesion such as acute trauma) were enrolled consecutively. Clinical data were prospectively collected, before the results of laboratory examination were available.

Laboratory tests followed the procedures recommended by WHO [2]. Dry swabs from ulcerative lesions were examined after ZN staining in Akonolinga Hospital laboratory on the same day. Another set of swabs was stored at 4°C on site and sent weekly to the reference laboratory in Yaounde (Centre Pasteur Cameroon), where, after pooling of the three samples for each lesion, ZN direct examination was repeated, followed by PCR and culture. Detection of *M*.*ulcerans* DNA from swabs was done by quantitative PCR (Taqman Assay) using oligonucleotide primer and Taqman probes from IS 2404 as previously described [8]. Swabs were homogenized in sterile water. For DNA extraction, 400 μl of sample was centrifuged at 3000 x *g* for 10 minutes. The pellet was washed twice with 800 μl of DNAse-free water per wash- After centrifugation at 3000 x *g* for 10 minutes, the recuperated pellet was suspended in 50 μl of 50 mM NaOH and heated at 95°C for 20 minutes to lyse the bacteria. DNA was purified with a QIAquick purification kit (Qiagen), according to the manufacturer’s recommendations. Culture was performed on Löwenstein-Jensen medium at 32°C, after decontamination using the N-acetyl-L-cysteine-sodium hydroxide method. Identification of *M*.*ulcerans* from culture was confirmed by combining culture characteristics and results of conventional IS2404 PCR after electrophoresis migration. Cultures were confirmed positive for *M*.*ulcerans* when they yielded a 515bp PCR product that lined up with positive control.

Statistical analyses were performed using the Stata/SE 12.1 (College Station, USA) and R 3.1.2 (R Core Team (2014). R: A language and environment for statistical computing. R Foundation for Statistical Computing, Vienna, Austria. URL http://www.R-project.org/). Results of the laboratory tests (ZN, PCR, culture) were modeled through a latent class model. The latent class model [9] hypothesizes the existence of an unobserved variable to explain the relationship among a set of observed variables. In this study, the observed variables were the laboratory tests and the unobserved variable represented the true status on the disease (the latent classes). Assuming that the tests are independent given the latent class, the probability of observing a particular pattern of responses to the tests follows a multivariate Bernoulli distribution. Parameters of the model were the prevalence of each latent classes and the sensitivity and specificity of each laboratory test. Estimation of these parameters was obtained from the maximum-likelihood estimation using an expectation-maximization algorithm. The hypothesis of conditional independence of the tests was assessed by adding a random parameter to model the conditional dependence among the tests. Bayesian Information Criteria (BIC) was used to assess model fitting. Clinical diagnosis was not included in the latent class model, to ensure independence with clinical variables assessed as predictors. Patients with missing laboratory results were excluded from the latent class model. Membership probabilities of each individual to each latent class were calculated according to test results. Patients were then classified into a high or a low BU likelihood, according to the largest membership probability. This classification was used as reference standard, or outcome of the prediction model.

The clinical variables to be explored as predictors were selected based on pre-existing literature and priority ranking by two experts external to the study. Because one patient could be affected by more than one lesion, variables at patient and lesion level were first analysed separately. Demographic characteristics and clinical variables associated with a high BU likelihood in the univariate analysis (p<0.20) were included in a multivariate logistic model. In the multivariate model, we only used lesion characteristics of the largest lesion (one lesion per patient). There were not enough cases with multiple lesions to warrant a hierarchical model. The few missing data among predictor variables were not imputed and were included as separate categories. After adjustment, variables still associated with BU at an OR>3/2 (1.5) or <2/3 (0.67) were selected for the score. The number of points attributed to each item in the score was the doubled value of the rounded-off (to the nearest 0.5) coefficient in the regression model. Discrimination of the model was assessed by using the area under the receiver operator characteristic curve. Model comparison was based on AUC (c-statistic) and final patient classification compared with BU category based on the latent class analysis. Post-estimation including Hosmer-Lemeshow goodness-of-fit, leverage and Pregibon’s Dbeta were then performed to check the validity of the model. Internal validation was performed using bootstrapping techniques to obtain a corrected c-statistic [5].

Sensitivity, specificity and predictive values were calculated for each cut-off of the score. Model calibration was based on predefined predictive values: choice of a cut-off for BU treatment was predefined as a positive predictive value above 70%. To exclude BU, the negative predictive value had to be above 95% (with a 95%CI above 90%). The algorithm based on the selected cut-offs was theoretically and retrospectively applied on the patients included in the study to estimate its performance and the proportion of patients not requiring a PCR.

In sensitivity analyses we compared this model with a more restrictive model keeping variables associated with BU with an OR>2 or <0.5, assessed the performance of the score by study period and mode of recruitment (referred or spontaneously presenting patient). Comparison was based on total area under the ROC curve and patient classification.

No formal sample size calculation was performed. Based on previous studies using latent class analysis [10] on sample of about 300 individuals, and given an annual expected number of 120 confirmed and 250 suspect Buruli cases, we decided on a consecutive recruitment over a period of two years, aiming at a sample of about 500 patients.

### Ethics statement

Ethical approval was given by the National Ethics Committee of Cameroon, the Central Commission on Human Subject Research Ethics of the Geneva University Hospital, and the Ethics Committee of Médecins Sans Frontières. The study was further authorized by the Ministry of Health, in the framework of the National Buruli Control Program, as well as from the health authorities of the Akonolinga District and Akonolinga Hospital administration. All patients provided written informed consent.

## Results

Between October 2011 and December 2013, 367 patients were included in the study, out of 447 screened, and 364 were finally analyzed (3 secondary exclusions due to missing clinical data), corresponding to 422 lesions, of which 381 were ulcerative (90.3%). Detailed patient flow is presented elsewhere [11]. There were more inclusions during the first half of the study period compared to the second half (215 vs. 110). Because ulcerative and non-ulcerative lesions had different clinical characteristics, the prediction model was based on the 325 patients with 379 ulcerative lesions and available laboratory results (missing for two patients).

Median age was 37 years (range 0 to 87), with 28.9% aged up to 20 years, 26.5% from 20 to 40 years, and 44.6% above 40 years (Table 1). Overall 212 (65%) were males and 63 (19.4%) were HIV-positive, with a median CD4 count of 362 (IQR 210–653; 12 missing CD4 count). In terms of other comorbidities, hypertension was confirmed in 4 cases (1.2%) and suspected in another 9 (2.8%); diabetes was confirmed and suspected in 7 (2.2%) and 22 (6.8%) cases, respectively. Sickle cell disease was confirmed in 6 (1.8%) patients. By severity grading according to WHO classification for Buruli ulcer, patients were of category I, II or III in 41.5%, 30.5% and 28.0%, respectively. Demographic and clinical characteristics are shown in Table 1.

**Table 1:** Univariate analysis of variables associated with BU likelihood, Akonolinga, Cameroon.

**Patient characteristics**	Total		High BU likelihood		Low BU likelihood		
	(N = 325)		(N = 51)		(N = 274)		
	n	%	n	%	n	%	p-value
Age							<0.001
≤ 20 years	94	28.9	35	68.6	59	21.5	
> 20 and ≤ 40 years	86	26.5	10	19.6	76	27.7	
> 40years	145	44.6	6	11.8	139	50.7	
Sex							0.008
Male	212	65.2	25	49.0	187	68.3	
Female	113	34.8	26	51.0	87	31.8	
Other treatments received							
Local	211	64.9	28	54.9	183	66.8	0.102
Systemic	223	68.6	27	52.9	196	71.5	0.009
History of trauma	117	36.0	13	25.5	104	38.0	0.089
Oedema							0.157
None	147	45.9	24	47.1	123	45.7	
Perilesional	101	31.6	21	41.2	80	29.7	
Of the affected limb	62	19.4	6	11.8	56	20.8	
Both lower limbs	10	3.1	0	0.0	10	3.7	
Missing	5				5		
Duration of present episode (in weeks)							
Missing	7						
Median (IQR)	24	5–104	8	4–28	28	5–108	<0.001
**Lesion characteristics**	Total		High BU likelihood		Low BU likelihood		
	(N = 379)		(N = 59)		(N = 320)		
	n	%	n	%	n	%	p-value
Localisation							0.001
Upper limb	35	9.2	13	22.0	22	6.9	
Lower limb	322	85.0	42	71.2	280	87.5	
Trunk	22.0	5.8	4	6.8	18	5.6	
Lesion size							0.075
≤ 5cm	161	42.5	33	55.9	128	40.0	
>5–≤ 15cm	151	39.8	18	30.5	133	41.6	
>15cm	67	17.7	8	13.6	59	18.4	
Hyposensitivity at lesion site	10	2.6	3	5.1	7	2.2	0.193
Induration (recode consist)	118	31.4	14	23.7	104	32.8	0.168
Locoregional adenopathy	89	23.5	7	11.9	82	25.6	0.022
Pain at rest	218	57.7	26	44.1	192	60.2	0.021
Undermining	133	35.1	37	62.7	96	30.0	<0.001
Characteristic smell (6 missing)							<0.001
Yes	39	10.5	17	28.8	22	7.01	
No	334	89.5	42	71.2	292	92.99	
Color (more than one possible answer)							
Green (pus)	88	23.2	19	32.2	69	21.56	0.075
Black (necrosis)	98	25.9	18	30.5	80	25	0.375
Yellow (fibrin)	296	78.1	54	91.5	242	75.63	0.007
Red (granulation)	309	81.5	41	69.5	268	83.75	0.01
Pink (epithelization)	66	17.4	7	11.9	59	18.44	0.221

**Fig 1:**
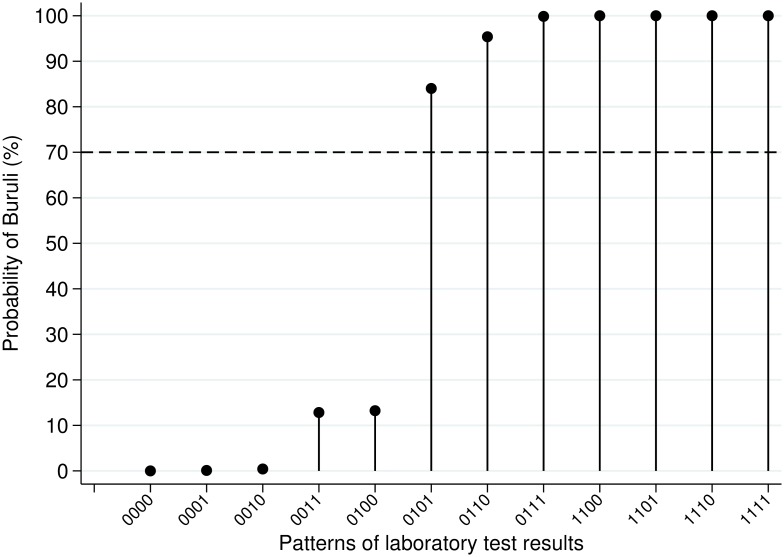
Estimated probability of Buruli ulcer according to pattern of laboratory test results in latent class model (1 = positive result, 0 = negative result; test order ZN CPC, PCR, culture, ZN Akonolinga. Dashed line: 70% probability of Buruli ulcer (predefined treatment threshold).

Proportion of positive laboratory tests for ulcerative lesions (N = 379) varied between 7.9% for culture and 22.2% for PCR. ZN was positive for 17.4% in Akonolinga and 10.6% in CPC. The estimated BU prevalence from the latent class model was 16.1% (95%CI 12.4–20.7%). Estimated sensitivity went from 46% for culture to 100% for PCR, with intermediate results for ZN (65% and 72% in Yaounde and Akonolinga, respectively; Table 2). Specificity was best for ZN Yaounde (100%) and culture (99%), followed by PCR and ZN Akonolinga (93%). Patients could be clearly discriminated into two groups according to their pattern of laboratory test results: a high BU likelihood (probability >0.8) and a low BU likelihood (probability <0.15) Fig 1).

**Table 2:** Parameter estimates from latent class analysis (N = 325).

BU prevalence (%)	16.1	(12.4–20.7)		
	Sensitivity		Specificity	
ZN Akonolinga	0.72	(0.60,0.85)	0.93	(0.90,0.96)
ZN CPC*	0.65	(0.51,0.80)	1.00	(1.00,1.00)
PCR	1.00	(0.97,1.00)	0.93	(0.89,0.96)
Culture	0.46	(0.33,0.59)	0.99	(0.98,1.00)

*CPC:Centre Pasteur Cameroun

In the univariate analysis, the following patient variables were found associated with BU likelihood at a p-value <0.20: duration of the episode, topical or systemic treatment (oral or parenteral) received previously, history of trauma, type of oedema, age, and gender (Table 1). Variables at lesion level associated with BU likelihood were localization, lesion size, hyposensitivity, induration, locoregional adenopathy, pain at rest, undermining, characteristic smell, green (purulent), yellow (fibrinous), and red (tissue granulation) color. The complete results of the univariate analysis are detailed in the S1 Table. None of the comorbidities (HIV, diabetes, hypertension) was found to be associated with BU.

**Fig 2:**
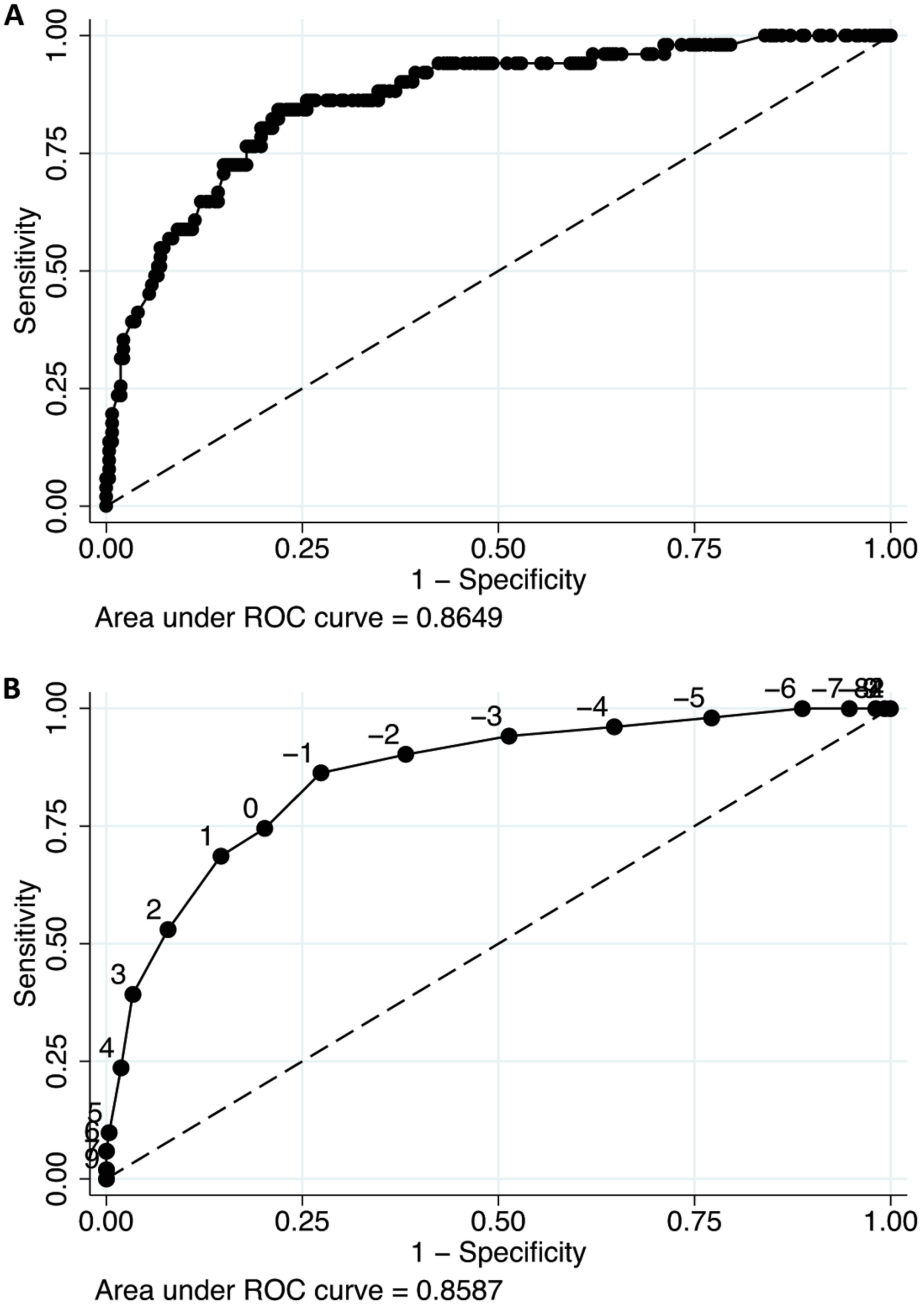
ROC curve of multivariable prediction model (A) and Buruli score (B).

**Fig 3:**
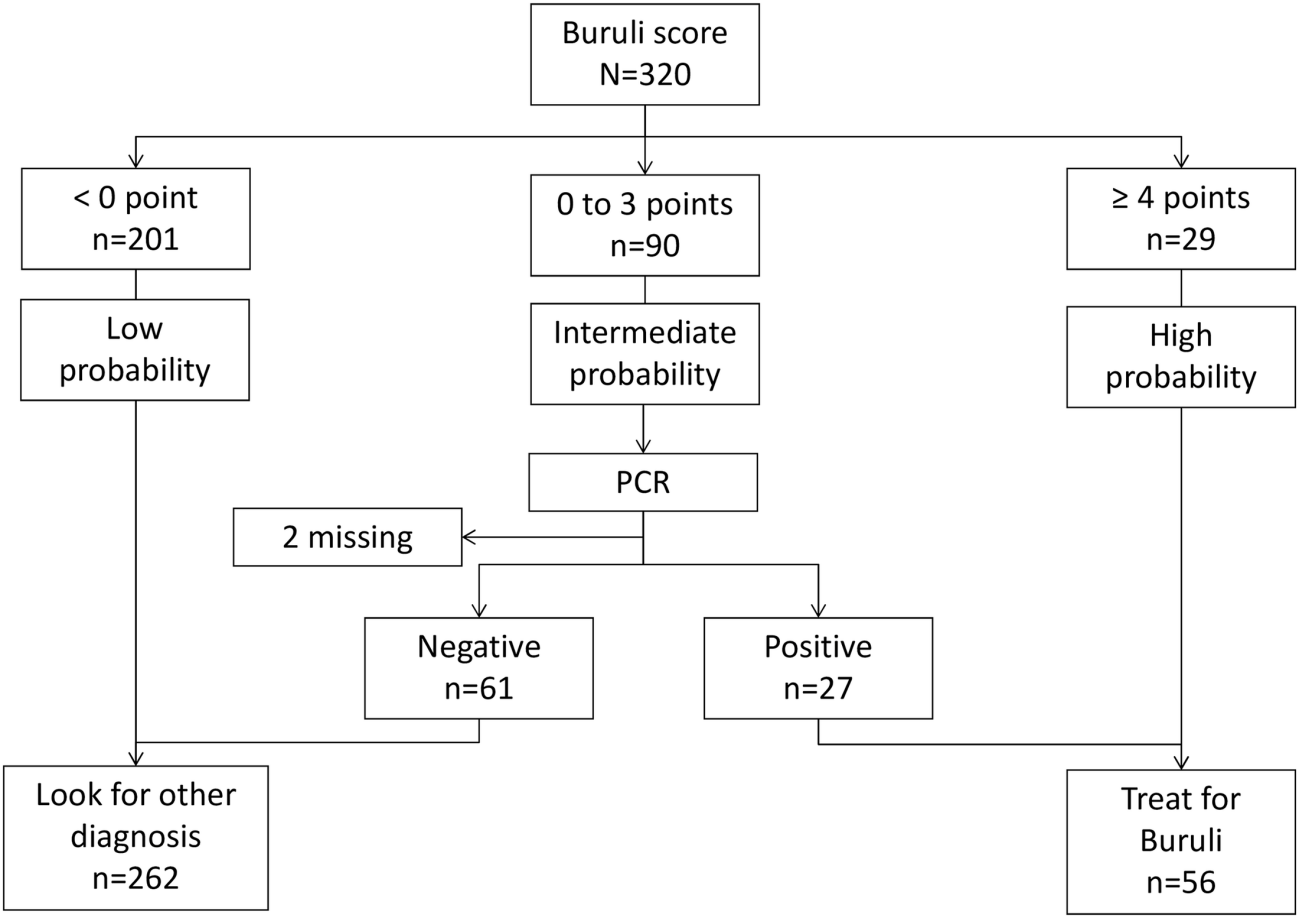
Algorithm based on Buruli score applied to the patients included in the study.

After adjustment with the other variables in the model, the following variables were included in the Buruli score based on an odds ratio >1.5 or <0.67 (Table 3): characteristic smell (+3 points), yellow color (+2), female gender (+2), undermining (+1), green color (+1), lesion hyposensitivity (+1), pain at rest (-1), lesion size above 5cm (-1), locoregional adenopathy (-2), age above 20 up to 40 years (-3), and age above 40 years (-5). The Buruli score had an area under the ROC curve (AUC) of 0.86 (95%CI 0.82–0.89) using the outcome of the latent class model as reference, similar to the AUC of the full multivariate model (Fig 2). The cut-off to reasonably exclude BU was set at scores < 0 (NPV 96.5% 95%CI 93.0–98.6). The treatment threshold was set at a cut-off ≥4 points (PPV 69.0, 95%CI 49.2–84.7). Patients with scores between 0 and 3 had an intermediate probability of BU and would need to be tested further by PCR. Using the algorithm on the patients included in the study (Fig 3), 56 patients would have been treated for BU, including 12 “false-positives” using the outcome of the latent class model as reference (specificity 95.5%; 95%CI 92.3–97.7). Seven BU cases would have been missed by the algorithm (sensitivity 86.3%; 95%CI 73.7–94.3). Three of them were HIV-positive, one had diabetes and another diabetes suspicion. Overall, PCR would have been performed in 27.7% (90/320) of the patients.

**Table 3:** Numbers and percentages according to BU likelihood, crude odd ratios, adjusted odds ratios and points attributed in Buruli score of items associated with BU probability.

	High BU likelihood (N = 51)	Low BU likelihood (N = 274)	Crude Odds Ratio	Adj Odds Ratio*	Points in Buruli score
	n (%)	n (%)	OR (95%CI)	aOR (95%CI)	
Characteristic smell	14 (27.5)	20 (7.5)	4.7 (2.1–10.3)	4.7 (1.7–12.9)	+3
Yellow color (fibrin)	46 (90.2)	207 (75.6)	3.0 (1.1–7.8)	3.3 (1.2–9.7)	+2
Female gender	26 (51.0)	87 (31.8)	2.2 (1.2–4.1)	2.3 (1.1–4.8)	+2
Undermining	33 (64.7)	87 (31.8)	3.9 (2.1–7.4)	1.9 (0.9–4.0)	+1
Green color (pus)	17 (33.3)	62 (22.6)	1.7 (.89–3.3)	1.7 (.76–4.0)	+1
Lesion hyposensitivity	3 (5.9)	7 (2.6)	2.4 (.60–9.5)	2.1 (0.3–14.3)	+1
Pain at rest	19 (37.3)	158 (57.9)	.43 (.23–.80)	.52 (.24–1.13)	-1
Lesion size >5cm	22 (43.1)	161 (58.8)	.53 (.29–.97)	.67 (.31–1.47)	-1
Locoregional adenopathy	7 (13.7)	74 (27.0)	.43 (.19–1.0)	.41 (.15–1.08)	-2
Age > 20 and ≤ 40 years	10 (19.6)	76 (27.7)	.22 (.10–.48)	.28 (.11–.69)	-3
Age > 40 years	6 (11.8)	139 (50.7)	.07 (.03–.18)	.09 (.03–.25)	-5

Patients with missing values: 6 for variable “smell”, 1 for “pain”, omitted for odds ratio calculation.

* Adjusted for the other variables included in the Buruli score

Hosmer-Lemeshow goodness-of-fit showed that our model predicted well the observed data (p = 0.24). Pregibon’s leverage and delta beta plots did not show major influential observations on model fitting in the dataset. The internal bootstrapping validated c-statistic was 0.76 (from 0.86 initially). Excluding variables with smaller coefficients from the score (green color, undermining, hyposensitivity, pain at rest, and lesion size >5cm) tended to decrease the area under the curve (0.85, 95%CI 0.80–0.88) and affected patient classification. Study period and mode of recruitment did not affect score performance

## Discussion

Based on our analysis of clinical predictors of Buruli ulcer diagnosis, we developed a score combining ten clinical and demographic characteristics, which predicts a high, intermediate or low probability that a patient has Buruli ulcer. Patients with a high probability can be treated without waiting for further test results, while those with a low probability should first be evaluated and treated for other diseases. Only patients with an intermediate probability need to be investigated further by PCR.

Sensitivity of the algorithm is not perfect, resulting in some true Buruli ulcer cases being missed, especially patients with comorbidities such as HIV or diabetes with atypical lesions. However, sensitivity of the algorithm is better than basing diagnosis on ZN alone, which is often the only test available in remote settings. Sensitivities of the laboratory tests that we used in our study are much better than results reported by previous studies, with PCR reaching 100% sensitivity, despite the fact that we did not use transport medium. However, comparisons between studies are very limited in the absence of standardization of the reference standards used. Also, we based patient classification (high vs. low BU likelihood) on results of laboratory tests only. We cannot exclude that some true Buruli cases had no positive laboratory results, and therefore were misclassified. This could have overestimated the performance of the laboratory tests. Our study was the first to use latent class analysis to build a reference for Buruli ulcer. This allowed us to construct an objective reference standard, not affected by clinical judgment. Although we acknowledge that our approach may also have diluted the effect of some clinical variables especially those associated with test-negative BU, we believe it would not have had an important impact on the final score.

Age was the strongest predictor of Buruli ulcer in our data, and therefore has an important weight in the score. Above 20 and particularly above 40 years of age, one needs strong other clinical arguments to suspect BU, especially among males which consult more frequently for other diseases. Our data confirm that BU is mainly a paediatric disease in Africa, even if older age groups can be affected, which is consistent with data from Ghana and Benin [12, 13] but different from Australia where the elderly are mostly affected [14], probably reflecting differences in exposure and possibly development of immunity. Currently the score should not be used in settings with a different age structure.

Interestingly, HIV infection was not found to be associated with BU diagnosis among patients with skin lesions. Also, we found that some clinical parameters that were thought typical of Buruli were in fact just typical of skin problems in an African context: indeed long duration of symptoms, localization on the lower limbs, and large lesions were found more often in non-BU than in BU lesions.

Some clinical items included in the Buruli score need further standardization. While yellow or green color, undermining, pain, size and locoregional adenopathy are relatively easy to determine, characteristic smell and lesion hyposensitivity may be more observer-dependent. In our study lesion hyposensitivity was defined as a diminished sensitivity to the touch within the ulcer but we did not use a standardized tool such as a monofilament. While painlessness is classically described in BU [15] and has been attributed to nerve damage by mycolactone [16, 17], hyposensitivity is more rarely reported. The underlying pathogenesis might be similar to painlessness, but this cannot be assessed by the same animal models. A characteristic Buruli smell, according to the clinicians, was described as a strong smell, like the smell of rotten fish, cassava or cheese, mixed with smell of pyocyanic bacteria. Indeed, both green color and characteristic smell were associated with BU, which could point towards *Pseudomonas* superinfection. This unpleasant smell has been described as one of the main stigmatizing factor among patients suffering from BU [18].

Because of the limitations of clinical diagnosis, we chose a very broad definition of “BU suspect” as inclusion criteria, in order not to select only BU patients corresponding to classical cases. Therefore, only a limited number of patients were finally classified as BU. The final number of included patients was below our expected sample size because of a decrease in number of Buruli cases in Akonolinga, as seen throughout West and Central Africa, although there is no clear explanation for this phenomenon. As a result, some predictors such as hyposensitivity only had few events and would need further validation to confirm their usefulness in a score. This decrease may also reflect a shift in patient population over the course of the study, although our sensitivity analysis was reassuring, with no evidence for a period effect. Besides, the difference between the apparent and bootstrap adjusted c-statistic confirms some degree of overfitting and reinforces the need for external validation. Still, even after adjustment, the c-statistic indicates good model performance. We hypothesized conditional independence between the laboratory tests included in the latent class model, although this can be questioned. However, the addition of a random parameter to model the conditional dependence between the tests did not show any improvement of model fitting.

### Conclusion

We developed a decisional algorithm based on a clinical score to assess the probability of BU infection among suspects. Applying the algorithm to the patients included in the study would have resulted in almost four times less PCR performed. After this first study on calibration, the Buruli score requires external validation before it can be recommended for wide use.

## Supporting Information

S1 Table Univariate analysis of variables potentially associated with BU likelihood, Akonolinga, Cameroon. (DOC)

S1 Checklist TRIPOD checklist for Prediction Model Development and Validation. (DOCX)

S1 Protocol Study protocol. (PDF)
