# Supplementary material for: The “Buruli Score”: Development of a Multivariable Prediction Model for Diagnosis of Mycobacterium ulcerans Infection in Individuals with Ulcerative Skin Lesions, Akonolinga, Cameroon
Source: PLoS Negl Trop Dis. 2016 Apr 5;10(4):e0004593. doi: 10.1371/journal.pntd.0004593 (PMC4821558; doi:10.1371/journal.pntd.0004593)
Supplement: S1 Table — (DOC) [file pntd.0004593.s001.doc]

Supplementary table. Univariate analysis of variables potentially associated with BU likelihood, Akonolinga, Cameroon.

| **Patient characteristics** | | Total |  | High BU likelihood | | Low BU likelihood | |  |
| --- | --- | --- | --- | --- | --- | --- | --- | --- |
|  |  | (N=325) |  | (N=51) |  | (N=274) |  |  |
|  |  | n | % | n | % | n | % | p-value |
| Age |  |  |  |  |  |  |  | <0.001 |
|  | ≤ 20 years | 94 | 28.9 | 35 | 68.6 | 59 | 21.5 |  |
|  | > 20 and ≤ 40 years | 86 | 26.5 | 10 | 19.6 | 76 | 27.7 |  |
|  | > 40years | 145 | 44.6 | 6 | 11.8 | 139 | 50.7 |  |
| Sex |  |  |  |  |  |  |  | 0.008 |
|  | Male | 212 | 65.2 | 25 | 49.0 | 187 | 68.3 |  |
|  | Female | 113 | 34.8 | 26 | 51.0 | 87 | 31.8 |  |
| Traditional treatment received | | 189 | 58.2 | 26 | 51.0 | 163 | 59.5 | 0.258 |
| Other treatments received | |  |  |  |  |  |  |  |
|  | Local | 211 | 64.9 | 28 | 54.9 | 183 | 66.8 | 0.102 |
|  | Systemic | 223 | 68.6 | 27 | 52.9 | 196 | 71.5 | 0.009 |
| History of trauma | | 117 | 36.0 | 13 | 25.5 | 104 | 38.0 | 0.089 |
| BU cases in the vicinity (2 missing) | | |  |  |  |  |  | 0.468 |
|  | No | 205 | 63.5 | 29 | 56.9 | 176 | 64.7 |  |
|  | Yes, same roof | 42 | 13.0 | 9 | 17.7 | 33 | 12.1 |  |
|  | Yes, same village | 76 | 23.5 | 13 | 25.5 | 63 | 23.2 |  |
| History of fever (1 missing) | | 26 | 8.0 | 6 | 11.8 | 20 | 7.3 | 0.284 |
| Oedema |  |  |  |  |  |  |  | 0.157 |
|  | None | 147 | 45.9 | 24 | 47.1 | 123 | 45.7 |  |
|  | Perilesional | 101 | 31.6 | 21 | 41.2 | 80 | 29.7 |  |
|  | Of the affected limb | 62 | 19.4 | 6 | 11.8 | 56 | 20.8 |  |
|  | Both lower limbs | 10 | 3.1 | 0 | 0.0 | 10 | 3.7 |  |
|  | Missing | 5 |  |  |  | 5 |  |  |
| Number of lesions | |  |  |  |  |  |  | 0.639 |
|  | One | 280 | 86.2 | 45 | 88.2 | 235 | 85.8 |  |
|  | More than one | 45 | 13.9 | 6 | 11.8 | 39 | 14.2 |  |
| Duration of present episode (in weeks) | | |  |  |  |  |  |  |
|  | Missing | 7 |  |  |  |  |  |  |
|  | Median (IQR) | 24 | 5 - 104 | 8 | 4 - 28 | 28 | 5 - 108 | <0.001 |
| **Lesion characteristics** | | Total |  | High BU likelihood | | Low BU likelihood | |  |
|  |  | (N=379) |  | (N=59) |  | (N=320) |  |  |
|  |  | n | % | n | % | n | % | p-value |
| Localisation | |  |  |  |  |  |  | 0.001 |
|  | Upper limb | 35 | 9.2 | 13 | 22.0 | 22 | 6.9 |  |
|  | Lower limb | 322 | 85.0 | 42 | 71.2 | 280 | 87.5 |  |
|  | Trunk | 22.0 | 5.8 | 4 | 6.8 | 18 | 5.6 |  |
| Lesion size |  |  |  |  |  |  |  | 0.075 |
|  | ≤ 5cm | 161 | 42.5 | 33 | 55.9 | 128 | 40.0 |  |
|  | >5 - ≤ 15cm | 151 | 39.8 | 18 | 30.5 | 133 | 41.6 |  |
|  | >15cm | 67 | 17.7 | 8 | 13.6 | 59 | 18.4 |  |
| Depth of the lesion | |  |  |  |  |  |  | 0.954 |
|  | No ulceration / superficial abrasion | 4 | 1.3 | 1 | 1.7 | 5 | 1.3 |  |
|  | Dermis | 69 | 21.6 | 11 | 18.6 | 80 | 21.1 |  |
|  | Hypodermis | 134 | 41.9 | 26 | 44.1 | 160 | 42.2 |  |
|  | Muscle, bone, tendon, joint | 113 | 35.3 | 21 | 35.6 | 134 | 35.4 |  |
| Suspicion of bone involvement | | 133 | 35.1 | 19 | 32.2 | 114 | 35.6 | 0.613 |
| Hyposensitivity at lesion site | | 10 | 2.6 | 3 | 5.1 | 7 | 2.2 | 0.193 |
| Warmth |  | 83 | 21.9 | 16 | 27.1 | 67 | 20.9 | 0.291 |
| Consistance (3 missing) | |  |  |  |  |  |  | 0.257 |
|  | Soft | 191 | 50.8 | 31 | 52.5 | 160 | 50.5 |  |
|  | Indurated | 118 | 31.4 | 14 | 23.7 | 104 | 32.8 |  |
|  | Infiltrated ("quilted") | 67 | 17.8 | 14 | 23.7 | 53 | 16.7 |  |
| Induration (recode consist) | | 118 | 31.4 | 14 | 23.7 | 104 | 32.8 | 0.168 |
| Local prurigo | | 113 | 29.8 | 17 | 28.8 | 96 | 30.0 | 0.855 |
| Locoregional adenopathy | | 89 | 23.5 | 7 | 11.9 | 82 | 25.6 | 0.022 |
| Pain assessment (1 missing) | |  |  |  |  |  |  |  |
|  | Pain at rest | 218 | 57.7 | 26 | 44.1 | 192 | 60.2 | 0.021 |
|  | Pain during wound dressing | 315 | 83.3 | 48 | 81.4 | 267 | 83.7 | 0.657 |
| Undermining | | 133 | 35.1 | 37 | 62.7 | 96 | 30.0 | <0.001 |
| Lesion edges (1 missing) | |  |  |  |  |  |  | 0.262 |
|  | Normal | 86 | 22.8 | 12 | 20.3 | 74 | 23.2 |  |
|  | Dry / keratinized | 66 | 17.5 | 6 | 10.2 | 60 | 18.81 |  |
|  | Macerated | 48 | 12.7 | 7 | 11.9 | 41 | 12.85 |  |
|  | Inflammatory | 178 | 47.1 | 34 | 57.6 | 144 | 45.14 |  |
| Characteristic smell (6 missing) | |  |  |  |  |  |  | <0.001 |
|  | Yes | 39 | 10.5 | 17 | 28.8 | 22 | 7.01 |  |
|  | No | 334 | 89.5 | 42 | 71.2 | 292 | 92.99 |  |
| Exsudate quantity | |  |  |  |  |  |  | 0.643 |
|  | None | 19 | 5.0 | 3 | 5.1 | 16 | 5 |  |
|  | Light | 254 | 67.0 | 36 | 61.0 | 218 | 68.13 |  |
|  | Moderate | 85 | 22.4 | 17 | 28.8 | 68 | 21.25 |  |
|  | Strong | 21 | 5.5 | 3 | 5.1 | 18 | 5.63 |  |
| Exsudate quality (if exsudate present, N=358) | | |  |  |  |  |  | 0.351 |
|  | Serous | 282 | 78.8 | 48 | 85.7 | 234 | 77.48 |  |
|  | Bloody | 25 | 7.0 | 2 | 3.6 | 23 | 7.62 |  |
|  | Purulent | 51 | 14.3 | 6 | 10.7 | 45 | 14.9 |  |
| Color (more than one possible answer) | | |  |  |  |  |  |  |
|  | Green (pus) | 88 | 23.2 | 19 | 32.2 | 69 | 21.56 | 0.075 |
|  | Black (necrosis) | 98 | 25.9 | 18 | 30.5 | 80 | 25 | 0.375 |
|  | Yellow (fibrin) | 296 | 78.1 | 54 | 91.5 | 242 | 75.63 | 0.007 |
|  | Red (granulation) | 309 | 81.5 | 41 | 69.5 | 268 | 83.75 | 0.01 |
|  | Pink (epithelization) | 66 | 17.4 | 7 | 11.9 | 59 | 18.44 | 0.221 |
