# Supplementary material for: The “Buruli Score”: Development of a Multivariable Prediction Model for Diagnosis of Mycobacterium ulcerans Infection in Individuals with Ulcerative Skin Lesions, Akonolinga, Cameroon
Source: PLoS Negl Trop Dis. 2016 Apr 5;10(4):e0004593. doi: 10.1371/journal.pntd.0004593 (PMC4821558; doi:10.1371/journal.pntd.0004593)
Supplement: S1 Checklist — (DOCX) [file pntd.0004593.s002.docx]

| **Section/Topic** | **Item** |  | **Checklist Item** | **Page** | **Text extracts** |
| --- | --- | --- | --- | --- | --- |
| **Title and abstract** | | | | |  |
| Title | 1 | D;V | Identify the study as developing and/or validating a multivariable prediction model, the target population, and the outcome to be predicted. | 1 | The “Buruli score”: development of a multivariable prediction model for diagnosis of *Mycobacterium ulcerans* infection in individuals with ulcerative skin lesions, Akonolinga, Cameroon |
| Abstract | 2 | D;V | Provide a summary of objectives, study design, setting, participants, sample size, predictors, outcome, statistical analysis, results, and conclusions. | 3 | See entire abstract |
| **Introduction** | | | | |  |
| Background and objectives | 3a | D;V | Explain the medical context (including whether diagnostic or prognostic) and rationale for developing or validating the multivariable prediction model, including references to existing models. | 5-6 | Because of the challenges of PCR-based diagnosis of BU, alternative methods should be explored. While rapid point-of-care tests are currently being developed (1), identifying which patients would truly benefit from a diagnostic test can reduce patient expenses and treatment delays for patients with a high probability of BU. Diagnostic scores derived from multivariable prediction models can be useful in clinical decision-making(8). These scores are based on diagnostic performance of various characteristics of medical history and clinical examination. For BU diagnosis, it has been proposed to combine ZN microscopy and PCR, only performing PCR in ZN-negative specimen, in order to reduce costs and keep diagnosis available at peripheral level for many patients (9). |
|  | 3b | D;V | Specify the objectives, including whether the study describes the development or validation of the model or both. | 6 | Therefore, we aimed to identify clinical predictors of BU diagnosis, in order to develop a multivariable prediction model (“Buruli score”), to assist clinicians working in resource-limited settings. |
| **Methods** | | | | |  |
| Source of data | 4a | D;V | Describe the study design or source of data (e.g., randomized trial, cohort, or registry data), separately for the development and validation data sets, if applicable. | 6 | Between 2011 and 2013, a prospective cohort study was conducted in Akonolinga Health District, central Cameroon, one of the endemic areas for BU in the country (10). |
|  | 4b | D;V | Specify the key study dates, including start of accrual; end of accrual; and, if applicable, end of follow-up. | 6 | Between 2011 and 2013, …  Clinical data were prospectively collected, before the results of laboratory examination were available. |
| Participants | 5a | D;V | Specify key elements of the study setting (e.g., primary care, secondary care, general population) including number and location of centres. |  | See accompanying paper, p5: In 2002 Médecins Sans Frontières, in collaboration with the Ministry of Health, began treating BU in the Akonolinga Health District, where the overall BU prevalence was 0.47% in 2007 [15]. |
|  | 5b | D;V | Describe eligibility criteria for participants. |  | All individuals presenting at Akonolinga District Hospital with a skin lesion suspect of new BU (either a nodule, a plaque, localized swelling and/or an ulcer) were enrolled consecutively after written consent. |
|  | 5c | D;V | Give details of treatments received, if relevant. |  | Not detailed |
| Outcome | 6a | D;V | Clearly define the outcome that is predicted by the prediction model, including how and when assessed. | 7 | Results of the laboratory tests (ZN, PCR, culture) were modeled through a latent class model. This model(11) hypothesizes the existence of a latent binary variable representing the true status of BU (the latent classes) and estimates BU prevalence, sensitivity and specificity of the tests included in the model. Clinical diagnosis was not included in the latent class model, to ensure independence with clinical variables assessed as predictors. Patients with missing laboratory results were excluded from the latent class model. Membership probabilities of each individual to each latent class were calculated according to test results. Patients were then classified into a high or a low BU probability, according to the largest membership probability. This classification was used as reference standard, or outcome of the prediction model. |
|  | 6b | D;V | Report any actions to blind assessment of the outcome to be predicted. | 6 | Clinical data were prospectively collected, before the results of laboratory examination were available. |
| Predictors | 7a | D;V | Clearly define all predictors used in developing or validating the multivariable prediction model, including how and when they were measured. | 7 | The clinical variables to be explored as predictors were selected based on pre-existing literature and priority ranking by two experts external to the study. |
|  | 7b | D;V | Report any actions to blind assessment of predictors for the outcome and other predictors. | 7 | Clinical data were prospectively collected, before the results of laboratory examination were available. |
| Sample size | 8 | D;V | Explain how the study size was arrived at. | 8 | No formal sample size calculation was performed. Based on previous studies using latent class analysis(12) on sample of about 300 individuals, and given an annual expected number of 120 confirmed and 250 suspect Buruli cases, we decided on a consecutive recruitment over a period of two years, aiming at a sample of about 500 patients. |
| Missing data | 9 | D;V | Describe how missing data were handled (e.g., complete-case analysis, single imputation, multiple imputation) with details of any imputation method. | 7 | The few missing data among predictor variables were not imputed and were included as separate categories. |
| Statistical analysis methods | 10a | D | Describe how predictors were handled in the analyses. |  | Because one patient could be affected by more than one lesion, variables at patient and lesion level were first analysed separately. Demographic characteristics and clinical variables associated with a high BU probability in the univariate analysis (p<0.20) were included in a multivariate logistic model. |
|  | 10b | D | Specify type of model, all model-building procedures (including any predictor selection), and method for internal validation. | 7-8 | Demographic characteristics and clinical variables associated with a high BU probability in the univariate analysis (p<0.20) were included in a multivariate logistic model. In the multivariate model, we only used lesion characteristics of the largest lesion (one lesion per patient). There were not enough cases with multiple lesions to warrant a hierarchical model. The few missing data among predictor variables were not imputed and were included as separate categories. After adjustment, variables still associated with BU at an OR>3/2 (1.5) or <2/3 (0.67) were selected for the score. The number of points attributed to each item in the score was the doubled value of the rounded-off (to the nearest 0.5) coefficient in the regression model. Discrimination of the model was assessed by using the area under the receiver operator characteristic curve. Post-estimation including Hosmer-Lemeshow goodness-of-fit, leverage and Pregibon’s Dbeta were then performed to check the validity of the model. Internal validation was performed using bootstrapping techniques to obtain a corrected c-statistic(8). |
|  | 10c | V | For validation, describe how the predictions were calculated. | NA |  |
|  | 10d | D;V | Specify all measures used to assess model performance and, if relevant, to compare multiple models. | 7-8 | Sensitivity, specificity and predictive values were calculated for each cut-off of the score. Model calibration was based on predefined predictive values: choice of a cut-off for BU treatment was predefined as a positive predictive value above 70%. To exclude BU, the negative predictive value had to be above 95% (with a 95%CI above 90%). The algorithm based on the selected cut-offs was theoretically and retrospectively applied on the patients included in the study to estimate its performance and the proportion of patients not requiring a PCR.  Model comparison was based on AUC (c-statistic) and final patient classification compared with BU category based on the latent class analysis. |
|  | 10e | V | Describe any model updating (e.g., recalibration) arising from the validation, if done. | NA |  |
| Risk groups | 11 | D;V | Provide details on how risk groups were created, if done. | 8 | Model calibration was based on predefined predictive values: choice of a cut-off for BU treatment was predefined as a positive predictive value above 70%. To exclude BU, the negative predictive value had to be above 95% (with a 95%CI above 90%). |
| Development vs. validation | 12 | V | For validation, identify any differences from the development data in setting, eligibility criteria, outcome, and predictors. | NA |  |
| **Results** | | | | |  |
| Participants | 13a | D;V | Describe the flow of participants through the study, including the number of participants with and without the outcome and, if applicable, a summary of the follow-up time. A diagram may be helpful. | 9 | See accompanying paper, figure 1.  Between October 2011 and December 2013, 367 patients were included in the study, out of 447 screened, and 364 were finally analyzed (3 secondary exclusions due to missing clinical data), corresponding to 422 lesions, of which 381 were ulcerative. Detailed patient flow is presented elsewhere (Toutous 2015, submitted). There were more inclusions during the first half of the study period compared to the second half (215 vs. 110). Because ulcerative and non-ulcerative lesions have different clinical characteristics, the prediction model was based on the 325 patients with 379 ulcerative lesions and available laboratory results (missing for two patients). |
|  | 13b | D;V | Describe the characteristics of the participants (basic demographics, clinical features, available predictors), including the number of participants with missing data for predictors and outcome. | 9 | Median age was 37 years (range 0 to 87), with 28.9% aged up to 20 years, 26.5% from 20 to 40 years, and 44.6% above 40 years (table 1). Overall 212 (65%) were males and 63 (19.4%) were HIV-positive, with a median CD4 count of 362 (IQR 210 – 653; 12 missing CD4 count). In terms of other comorbidities, hypertension was confirmed in 4 cases (1.2%) and suspected in another 9 (2.8%); diabetes was confirmed and suspected in 7 (2.2%) and 22 (6.8%) cases, respectively. Sickle cell disease was confirmed in 6 (1.8%) patients. By severity grading according to WHO classification for Buruli ulcer, patients were of category I, II or III in 41.5%, 30.5% and 28.0%, respectively. Demographic and clinical characteristics are shown in table 1. |
|  | 13c | V | For validation, show a comparison with the development data of the distribution of important variables (demographics, predictors and outcome). | NA |  |
| Model development | 14a | D | Specify the number of participants and outcome events in each analysis. | Table 1  Table 2  Table 3 | Denominators (N) are indicated in the tables |
|  | 14b | D | If done, report the unadjusted association between each candidate predictor and outcome. | Table 1 |  |
| Model specification | 15a | D | Present the full prediction model to allow predictions for individuals (i.e., all regression coefficients, and model intercept or baseline survival at a given time point). | Table 3 | Adjusted odd ratios are presented instead of logistic regression model coefficient. |
|  | 15b | D | Explain how to the use the prediction model. | 14 | The cut-off to reasonably exclude BU was set at scores < 0 (NPV 96.5% 95%CI 93.0 – 98.6). The treatment threshold was set at a cut-off ≥4 points (PPV 69.0, 95%CI 49.2 – 84.7). Patients with scores between 0 and 3 had an intermediate probability of BU and would need to be tested further by PCR. |
| Model performance | 16 | D;V | Report performance measures (with CIs) for the prediction model. | 14 | The Buruli score had an area under the ROC curve (AUC) of 0.86 (95%CI 0.82 – 0.89) using the outcome of the latent class model as reference, similar to the AUC of the full multivariate model (figure 2). |
| Model-updating | 17 | V | If done, report the results from any model updating (i.e., model specification, model performance). | 16 | Hosmer-Lemeshow goodness-of-fit showed that our model predicted well the observed data (p=0.24). Pregibon’s leverage and delta beta plots did not show major influential observations on model fitting in the dataset. The internal bootstrapping validated c-statistic was 0.76 (from 0.86 initially). Excluding variables with smaller coefficients from the score (green color, undermining, hyposensitivity, pain at rest, and lesion size >5cm) tended to decrease the area under the curve (0.85, 95%CI 0.80 – 0.88) and affected patient classification. Study period and mode of recruitment did not affect score performance. |
| **Discussion** | | | | |  |
| Limitations | 18 | D;V | Discuss any limitations of the study (such as nonrepresentative sample, few events per predictor, missing data). | 17,18 | Although we acknowledge that our approach may also have diluted the effect of some clinical variables especially those associated with test-negative BU, we believe it would not have had an important impact on the final score. […]  Some clinical items included in the Buruli score need further standardization.[….]  Because of the limited number of patients finally classified as BU, some predictors such as hyposensitivity only had few events and would need further validation to confirm their usefulness in a score. The final number of included patients was below our expected sample size, because of a decrease in number of Buruli cases in Akonolinga (MSF, unpublished data), as seen throughout West and Central Africa(2), although there is no clear explanation for this phenomenon. This decrease may also reflect a shift in patient population over the course of the study, although our sensitivity analysis was reassuring, with no evidence for a period effect. Besides, the difference between the apparent and bootstrap adjusted c-statistic confirm some degree of overfitting and reinforce the need for external validation. Still, even after adjustment, the c-statistic indicates good model performance. |
| Interpretation | 19a | V | For validation, discuss the results with reference to performance in the development data, and any other validation data. | 16-17 | Sensitivity of the algorithm is not perfect, resulting in some true Buruli ulcer cases being missed, especially patients with comorbidities such as HIV or diabetes with atypical lesions. However, sensitivity of the algorithm is better than basing diagnosis on ZN alone, which is often the only test available in remote settings. Sensitivities of the laboratory tests used in our study are much better than results reported by previous studies, with PCR reaching 100% sensitivity, despite the fact that we did not used transport medium. However, comparisons between studies are very limited in the absence of standardization of the reference standards used. Also, we based patient classification (high vs. low BU probability) on results of laboratory tests only. We cannot exclude that some true Buruli cases had no positive laboratory results, and therefore were misclassified. This could have overestimated the performance of the laboratory tests. |
|  | 19b | D;V | Give an overall interpretation of the results, considering objectives, limitations, results from similar studies, and other relevant evidence. | 17 | Conclusion  We developed a decisional algorithm based on a clinical score to assess the probability of BU infection among suspects. Applying the algorithm to the patients included in the study would have resulted in almost four times less PCR performed. After this first study on calibration, the Buruli score requires external validation before it can be |
| Implications | 20 | D;V | Discuss the potential clinical use of the model and implications for future research. | 16 | Based on our analysis of clinical predictors of Buruli ulcer diagnosis, we developed a score combining ten clinical and demographic characteristics, which predicts a high, intermediate or low probability that a patient has Buruli ulcer. Patients with a high probability can be treated without waiting for further test results, while those with a low probability should first be evaluated and treated for other diseases. Only patients with an intermediate probability need to be investigated further by PCR. |
| **Other information** | | | | |  |
| Supplementary information | 21 | D;V | Provide information about the availability of supplementary resources, such as study protocol, Web calculator, and data sets. |  | The data set underlying the findings of this study are available on request, in  accordance with the legal framework set forth by Médecins Sans Frontières (MSF)  data sharing policy (Karunakara U, PLoS Med 2013). The MSF data sharing policy  ensures that data will be available upon request to interested researchers while  addressing all security, legal, and ethical concerns. All readers may contact Ms.  Aminata Ndiaye (aminata.ndiaye@epicentre.msf.org) to request the data. |
| Funding | 22 | D;V | Give the source of funding and the role of the funders for the present study. | 19 | **Funding**  This work was supported by Médecins Sans Frontières, Operational Center Geneva. |

*Items relevant only to the development of a prediction model are denoted by D, items relating solely to a validation of a prediction model are denoted by V, and items relating to both are denoted D;V. We recommend using the TRIPOD Checklist in conjunction with the TRIPOD Explanation and Elaboration document.
