# Supplementary material for: The “Buruli Score”: Development of a Multivariable Prediction Model for Diagnosis of Mycobacterium ulcerans Infection in Individuals with Ulcerative Skin Lesions, Akonolinga, Cameroon
Source: PLoS Negl Trop Dis. 2016 Apr 5;10(4):e0004593. doi: 10.1371/journal.pntd.0004593 (PMC4821558; doi:10.1371/journal.pntd.0004593)
Supplement: S1 Protocol — (PDF) [file pntd.0004593.s003.pdf]

**Project title**	Sensitivity and specificity of a clinical score for diagnosis of *Mycobacterium ulcerans* infection in Akonolinga, Cameroon.
**Document**	Protocol
**Version**	English translation of version 18.07.2011.<br>- Revised by the N.A.C. Ethics Committee, 7 December 2010<br>- Corrections according to the National Ethical Committee of Cameroon of 11th of April 2011<br>- Corrections according to the Ethical Review Board of Médecins Sans Frontières of 27th of June 2011 and 7th of July 2011.
**Protocol developers**	Yolanda Müller[1], Mathieu Bastard[1], Muriel Rabilloud[2], Serge Maturin Kaboré[3], Earnest Njih Tabah[4], Eric Comte[5], Elizabeth Tchanz[6], Fabienne Nackers[1], Laurence Trellu[6], Jean-François Etard[1]
**Collaborating institutions**	1 Epicentre<br>2 Hospices civils de Lyon<br>3 Médecins Sans Frontières - Cameroon<br>4 National Buruli Ulcer Control Program, Ministry of Public Health, Cameroon<br>5 Médecins Sans Frontières - Switzerland<br>6 University Hospitals of Geneva<br>7 Centre Pasteur Cameroon
**Financed by**	MSF-CH

Summary

Title	Sensitivity and specificity of a clinical score for diagnosis of *Mycobacterium ulcerans* infection: latest update 22.09.2010
Hypothesis/ Objective	To establish a decision tree based on criteria from patient history, clinical examination, and laboratory tests for the diagnosis of skin lesions in endemic area for Buruli Ulcer.<br>To establish within the decision tree a diagnostic score based on a list of clinical data recorded during patient history and clinical examination.<br>The decision tree is intended for low-income countries with a high prevalence of the Buruli Ulcer, where health professionals have little access to laboratory capacities.
Objective	Optimisation of diagnosis of *Mycobacterium ulcerans* infection (*M. ulcerans*) for low-income countries
Location	Buruli Project MSF-CH, Akonolinga, Cameroon
Proposed Partner Organisations	University Hospitals of Geneva (HUG)<br>Hospices Civils de Lyon<br>Cameroon's Ministry of Health<br>Centre Pasteur Cameroon<br>Epicentre
Context	The largest number of *M. ulcerans*-infected cases in Cameroon is found along the Nyong River in the districts of Ayos and Akonolinga. Since 2002, Médecins Sans Frontières (MSF), in collaboration with the Ministry of Health, have been treating patients infected with *M. ulcerans* in the district of Akonolinga.
Background	In most cases, patients infected with *M. ulcerans* present with ulcerated skin lesions. Lesions are mainly located on the limbs though they can affect any part of the body.<br><br>These ulcers can be mistaken for other types of tropical skin diseases. Among the most common are vascular or diabetic ulcers, drepanocytosis, yaws, ecthyma, phagedenic ulcers or all other infectious ulcers.<br><br>The diagnosis of *Mycobacterium ulcerans* infection can benefit from laboratory confirmation from microscopic examination using Ziehl-Neelsen (ZN) staining technique, culture, PCR or histopathological tests. Unfortunately, histopathology, PCR, and culture are not easily accessible. The ZN method is the only test that can easily be conducted in endemic areas; however, the test has a low sensitivity rate (60%) and is not available everywhere.<br><br>Clinical examination remains the key factor in differentiating skin ulcers based on what causes them. Care providers working in peripheral health centres, however, may have limited knowledge in

	dermatology necessary for an accurate clinical diagnosis.<br><br>Using scores based on the patient's medical history and clinical examination can help with the clinical decision-making. For example, a the "Crofton score" chart (based on eleven criteria) or the Keith Edwards scoring system (based on eight criteria) are used in the diagnosis of tuberculosis in children to determine the need for treatment or not.<br><br>Establishing such a score chart as an aid to clinical decision-making could prove useful when having to diagnose a wound suspected to be *M. ulcerans* infection. It should be included in a decision tree allowing the care provider to use logical reasoning when faced with a skin lesion, whether ulcerative or non-ulcerative, using patient history, clinical tests and laboratory tests.
Methodology	Step 1: Clinical experts to define a set variables that would be useful in the medical diagnosis of *M. ulcerans* infection.<br>Step 2: Create a database of all patients suspected of infected with *Mycobacterium ulcerans* and include the variables found in all suspected cases.<br>Step 3: Develop a prediction model based on collected data.<br>Step 4: Validate the prediction model.<br><br>Reference standard for diagnosis of the infection:<br>A latent class model shall be used in the absence of a good reference standard in the diagnosis of *M. ulcerans* infection. The model will combine all available findings from various diagnostic tests to generate a probability to *M. ulcerans* infection. The probability obtained from the model will be used to build the clinical score.
Benefits	Optimisation of the diagnosis of *M. ulcerans* infection and, consequently, better treatment for patients and a better allocation of resources
Risks	Risks related to the skin biopsy.
Research duration	2 years
Responsibility	Epicentre: Drafting of research protocol, study implementation, follow-up, analysis of data, and publication.<br>MSF-OCG: Liaison between the different partners, collaboration in drawing up the protocol and interpreting findings.<br>MSF Project: Logistical and administrative support, request for ethics committee in Cameroon, contact with partner organisations in Cameroon, in charge of patients and data collection.<br>HUG: Technical support in dermatology for assessment of lesions and laboratory quality control.

	Hospices Civils de Lyon: Methodology support in the development of protocol and statistical support for data analysis

Table of Contents

Summary ... 2
Abbreviations and Acronyms ... 6
1.1 *Mycobacterium ulcerans* ... 7
1.2 Diagnostic Scores ... 9
1.3 *M. ulcerans* Infection in Cameroon ... 10
1.4 Akonolinga ... 10
2 Goals of the study ... 11
Main objective: ... 11
3 Material and methods ... 11
3.1 Design of the study ... 11
3.2 Place of investigation ... 11
3.3 Target population ... 12
3.4 Definition of cases ... 12
3.5 Criteria for inclusion ... 12
3.6 Exclusion criteria ... 12
3.7 Patient Recruitment ... 12
3.8 Sampling ... 12
3.9 Data Collection ... 12
3.10 Diagnostic tests ... 12
3.10.1 Diagnostic tests for infection with *M. ulcerans* ... 12
3.10.2 Other laboratory tests ... 13
3.10.3 Quality Control ... 13
3.11 Clinical opinion ... 14
3.12 Patient Monitoring ... 14
3.13 Statistical analysis. ... 14
4 Authorization and Consent ... 16
5 Analysis of risks and benefits ... 16
6 Financing ... 17
7 References ... 17
7 Appendix ... 19

Abbreviations and Acronyms

CPC	Centre Pasteur Cameroon
MSF	Médecins Sans Frontières
MSF-CH	Médecins Sans Frontières, Switzerland
NA	Non-applicable
FNA	Fine Needle Aspiration
PCR	Polymerase Chain Reaction
Se	Sensitivity
Sp	Specificity
BU	Buruli Ulcer
ZN	Ziehl-Neelsen

1 Introduction

1.1 *Mycobacterium ulcerans*

Infection by *M. ulcerans* is more commonly know as Buruli Ulcer (BU), although the disease is not always ulcerative, and is among the neglected diseases [1]. It manifests itself as skin lesions which, without proper treatment, can lead to mutilation and extensive tissue damage. Buruli Ulcer has been reported from 30 countries in Africa, the Americas, Asia and the Western Pacific. West Africa has the highest number of cases, found mainly in Benin, Ivory Coast, and Ghana. The disease is concentrated mainly along certain rivers and marshy areas in the affected countries.

M. ulcerans infection generally begins with a localised, itchy skin lesion evolving into localized (nodule) or diffused (oedema) swelling, or as an indurated plaque. The lesions are generally not painful. This may be due to the immunosuppressive properties of the *M. ulcerans* toxin, mycolactone. Over time, the lesions progressively develop into craters that result in potentially massive ulcers with undermined edges. *M. ulcerans* can also cause bone infection with serious osteoarticular consequences, during active stages of the disease or as sequelae. Without proper treatment, however, the scarring process can lead to contracture deformities and limited movement of the limbs [2].

Various forms of BU can be mistaken for other types of ulcers found in tropical areas. Among the most common are vascular or diabetic ulcers, drepanocytosis, yaws, ecthyma, phagedenic ulcers, chronic Herpes or all other infectious ulcers, as well as skin cancers. Leprosy is among the neurogenic ulcers. Differential diagnosis of non-ulcerative lesions mainly includes bacterial infections (cellulitis, hypodermitis, tuberculosous abscess, parasitosis or mycosis). Also included are other causes of nodules such as lipoma and or rare yet dreadful malignant tumours (sarcoma, lymphoma).

Table 1. Differential diagnoses of various forms of Buruli Ulcer, WHO Manual [3].

Papule	Nodule	Plaque	Oedema	Ulcer
Insect bites	Cyst	Leprosy	Cellulitis	Tropical phagedenic ulcer
Pimple	Lipoma	Cellulitis	Elephantiasis	Venous ulcer
Herpes	Onchocercoma	Mycosis	Actinomycosis	Leishmaniasis
Granuloma annulare	Boil	Psoriasis	Necrotizing fasciitis	Neurogenic ulcer
Psoriasis	Lymphadenitis	Haematoma	Osteomyelitis	Yaws
Pityriasis	Mycosis	Insect bites	Onchocercoma	Squamous cell carcinoma

Note: Infection caused by other mycobacterial organisms can be mistaken for any of the above.

Table 1 Differential diagnoses of various forms of Buruli ulcer

Cases of painless ulcerated plaques with undermined edges are relatively easy to diagnose clinically in endemic regions; the difficulty lies in diagnosing lesions in their early stages or located in areas where vascular ulcers are typical. The differential clinical diagnosis should also be broken down into age groups. For example, vascular ulcers are rare in children. In a retrospective study carried out in Ghana, the specificity of clinical diagnosis was estimated to be 94% [4]. Nevertheless, without laboratory testing to confirm the findings, diagnostic errors are probably highly under-estimated.

Laboratory testing to confirm diagnosis ensures that proper treatment be proposed and prevents the misuse of antibiotics. The technique most common used on-site in search of alcohol-resistant bacilli is the direct examination by microscopy using Ziehl-Neelsen stained smear from an ulcerated lesion. The sensitivity of this method, however, is relatively low at approximately 40%.[5,6] The same staining technique can be used for non-ulcerated plaques using fine needle aspiration.[7] Culture of *M. ulcerans* is a procedure with sensitivity rate of 20–60% [6]. It takes several weeks to obtain results from this test, which can unfortunately show a false negative result if the sample was taken after antibiotic treatment had begun [5]. Polymerase Chain Reaction for insertion sequence IS2404 is becoming increasingly used [6]. This test is very specific and is currently the most sensitive diagnostic method. [5]. The PCR technique is performed using lesion swabs, biopsy specimens or fine needle aspirations [8]. Save for its cost, PCR remains a method that is difficult to implement in rural areas where *M. ulcerans* is endemic. Histopathology is rarely used in the field, though sensitivity and specificity are quite high. The method requires adapted tools for sample collection as well as specific know-how in both preparing and interpreting the slides. Table 1 is a summary of various sensitivities and specificities using different tests, according to the most recent studies.

Tableau 1. Sensitivity for various for diagnostic testing for *M. ulcerans* infection

Lesion	Specimen	Test	Sensitivity (95%IC)	Gold standard	Area	Reference
Non-ulcerative	Punch or surgery	PCR	66.7-93.5	At least 1 test + (ZN, PCR, culture)	Ghana	[5]
		ZN	40.0-85.7			
		Culture	0.0-70.8			
	Punch or surgery	PCR	88.2	Definition as per WHO *	Benin	[9]
		ZN	58.8			
		Culture	58.8			
	FNA	PCR	88.2			
		ZN	64.7			
		Culture	41.2			
	Surgery	ZN	40.3	Clinical diagnosis	Ghana	[10]
		ZN + PCR	65.7			
Ulcerative	Smear	PCR	72.2-89.9	At least 1 test + (ZN, PCR, culture)	Ghana	[5]
		ZN	23.1-67.8			
		Culture	3.0-57.4			
	Punch or surgery	PCR	44.4-67.8			
		ZN	33.9-38.9			
		Culture	12.5-23.4			
	Punch or surgery	PCR	84.3	Definition as per WHO *	Benin	[9]
		ZN	31.4			
		Culture	13.7			
	FNA	PCR	35.3			
		ZN	21.6			
		Culture	9.8			
	Smear	ZN	29.8	Clinical diagnosis	Ghana	[10]

		ZN + PCR	68.1			
All lesions	FNA	PCR	86 (72-94)	PCR on punch biopsy	Ghana	[8]
		ZN	44 (29-60)			
		Culture	26 (14-41)			
	Punch	ZN	42 (29-56)	Histology or culture + or ZN+ with compatible histology	Ghana	[6]
		Culture	49 (35-63)			
		PCR	98 (91-100)			
		Histology	82 (70-90)			

* Definition as per WHO: positive for at least two tests

In the absence of a good gold standard, most of the diagnostic tests use a composite reference standard when evaluating diagnostic tools, i.e., the presence of at least one or two positive tests (PCR, culture or direct examination). As such, sensitivity estimations provided are systematically over-evaluated since patients whose test results are all negative are not included when calculating sensitivity, while false positives are included in composite gold standards that use the evaluated test. In a study held in Ghana using clinical diagnosis as gold standard [10], only 52.6% of the 161 patients clinically diagnosed with BU had at least 2 tests return positive; 70.8% of the patients had at least one come back positive. However, the histology test of the 47 patients whose tests did not come back positive allowed 34 others diagnoses to be identified. Excluding the latter, sensitivity of one or two positive diagnostic tests were 89.7% and 66.9%, respectively. The specificity, however, is only rarely evaluated; most articles report only results from tests conducted on patients presumed to be Buruli, not from patients in whom the diagnosis has been excluded.

1.2 Diagnostic Scores

Diagnostic scores can be useful in clinical decision-making. Classic examples of this type of scoring are the Crofton Score for tuberculosis diagnosis in children or the Geneva Score for diagnosis of pulmonary embolism. These scores are based on diagnostic performance of various characteristics of medical history and clinical examination. They are considered as diagnostic “tests” and are thus each characterised by a certain sensitivity and specificity. In combining these different elements, a model can be built whose diagnostic performance may be combined to that of laboratory tests.

Figure 1. Example of a diagnostic score chart [11]:

Table 1. Scoring of the 8 Variables in the Original and Simplified Revised Geneva Score

Variable	Original	Simplified
Age >65 y	1	1
Previous DVT or PE	3	1
Surgery (under general anesthesia) or fracture (of lower limbs) within 1 mo	2	1
Active malignant condition (solid or hematologic, currently active or considered cured <1 y)	2	1
Unilateral lower-limb pain	3	1
Hemoptysis	2	1
Heart rate, beats/min		
75-94	3	1
≥95	2	1
Pain on lower-limb deep venous palpation and unilateral edema	4	1

Abbreviations: DVT, deep vein thrombosis; PE, pulmonary embolism.

1.3 *M. ulcerans* Infection in Cameroon

In Cameroon, *M. ulcerans* infection cases have been reported in 6 provinces, namely Adamaoua, Central, South, South-East, East and Extreme North. The district of Akonolinga was identified as a health district of high prevalence. In this district and the neighbouring district of Ayos, endemic areas are located along the Nyong River. In 2007, the overall prevalence was 0.47% for all cases of BU in the district [12].

1.4 Akonolinga

In 2002, Médecins Sans Frontières, in collaboration with the Ministry of Health in Cameroon, began treating patients diagnosed with *M. ulcerans* infection. Since then, almost 850 patients have been taken care of. The current diagnostic procedure lies mainly on analyses of Ziehl-Neelsen tests performed in Akonolinga and at the Centre Pasteur Cameroon in Yaoundé, as well as PCR tests conducted in Yaoundé. For ulcerated lesions, the specimens used are swabs; whereas fine needle aspiration, or testing of biopsy tissue in case of surgery, is used for non-ulcerative plaques.

In 2009, 104 patients were included into the programme [13], 96 of which were new cases, for a total of 328 suspected cases. The cases seen in Akonolinga itself corresponded to 189 suspected cases and 95 cases of Buruli (56 hospitalised and 39 ambulatory), the other cases having been seen in peripheral health centres. In peripheral areas, 139 cases have thus been seen, nine were treated directly in the peripheral health centres, and the others were referred to Akonolinga (2 transfers). During the first half of 2010, 130 suspected cases were seen and 77 new cases were diagnosed. Between 120 and 160 new cases per 250-300 suspected cases are expected per annum.

Table 2. Origin of Buruli cases in the Akonolinga programme, MSF-CH, 2009.

	Akonolinga / hospital	Akonolinga / ambulatory	In periphery	Total
**2009**				
Suspected cases	189		139	328
New cases				
- hospitalised	56			56
- ambulatory	39		9	48

**Q1 2010**<br>-Suspected cases<br>- New cases	 58<br>32		 12<br>6	 58<br>32
**Q2 2010**<br>-Suspected cases<br>- New cases	 72<br>14	 <br>10	 <br>5	 72<br>29

The gender breakdown of patients (male/female) is balanced with a ratio of 1.0. Almost half the patients are over age 15. In Akonolinga, the median duration of symptoms prior to consultation was 8 weeks (quartile range of 4 to 20 weeks). La majority of patients had an ulcerative plaque (83%), with or without oedema. Seventy percent of the patients had their diagnosis confirmed by the laboratory, defined as at least one positive test (ZN or PCR). Between 25 and 30% of the patients benefited from surgery in addition to antibiotic treatment. The majority of the patients (59%) were hospitalised, with an average stay of 7 months. The treatment results show 83% of the patients had scarring without sequela, 5% had scarring with sequela, 3% were transferred, and 3% were lost to follow-up.

Within the scope of improving BU treatment in Akonolinga, MSF Switzerland has partnered with University Hospitals of Geneva (HUG) in order to refine the diagnosis with improved training in cutaneous lesions and by proposing specialised and field-adapted treatment. For the last four years, HUG experts (Dr A Calmy, Pr B Hirschel,, Dr H Vuagnat, Dr L Toutous Trellu) have actively been involved as part of the HUG humanitarian project grouping various objectives regarding BU, in partnership with MSF [14]. The project presented herein focuses on the diagnosis.

2 Goals of the study

Main objective:

The main objective of this study is to establish a score to support clinical decision making support when a *Mycobacterium ulcerans (M. ulcerans*) infection is suspected.

Secondary Objective:

- Establish a decision making tree based on criteria from the patient history, clinical examination and paraclinical tests for diagnosing skin lesions in endemic area of infection with *M. ulcerans.*

- Measure the interobserver variability of the clinical diagnosis of *M. ulcerans* infection

- Describe the differential diagnoses in cases of lesions suspect of *M. ulcerans* infection

3 Material and methods

3.1 Design of the study

Prospective diagnostic study

3.2 Place of investigation

Buruli project, MSF-CH, Akonolinga, Cameroon

3.3 Target population

Patients presenting to the MSF Akonolinga project for suspected infection with *Mycobacterium ulcerans*.

3.4 Definition of cases

A patient suspected of infection with *Mycobacterium ulcerans* is defined as a patient with a nodule, plaque, swelling and/or ulcer type skin lesion in an endemic area for *M. ulcerans.*

3.5 Criteria for inclusion

- Patients with new skin lesions suspected to be infections with *M. ulcerans* coming to the Akonolinga Center or to the decentralised teams part of the Akonolinga project.

3.6 Exclusion criteria

- History of medical or surgical treatment for infection with *M. ulcerans*

3.7 Patient Recruitment

All eligible patients will be approached for recruitment in the study

3.8 Sampling

Patients will be consecutively recruited during the study period (2 years). Based on about 120 confirmed cases of Buruli per year for 250 to 300 suspected cases, the final size of the sample will be approximately 500 to 600 suspected cases. Based on expert advice as well as existing literature(1;2), 300 subjects is the number generally recommended to be able to perform latent class analysis.

Field Code Changed

3.9 Data Collection

Data from the medical history and clinical examination will be collected using a questionnaire at the entrance examination (see patient medical record attached). Clinical lesions will be measured using a centimeter scale in the direction of the longest diameter through the center of the lesion. The results of laboratory tests will be noted in the questionnaire after obtaining results.

Data will be entered from the paper questionnaire into a database developed specifically for the project.

Each lesion will be photographed twice with two different magnifications at the initial consultation, with the presence of a centimeter scale in the image field.

3.10 Diagnostic tests

3.10.1 Diagnostic tests for infection with *M. ulcerans*

Overall, the procedures used in the Akonolinga project will follow the recommendations of the diagnostic manual published by WHO [3.]

Sample collection:
For ulcerative lesions, swabs are done in at least two different places around loose edges of the wound. Two smears are immediately sent to the Akonolinga laboratory to create two smears, whereas four other samples are stored in the refrigerator between 2 and 8 ° C, for subsequent shipment to the Centre Pasteur of Cameroon for direct examination (2 smears), mycobacteria culture and PCR.
For non-ulcerative lesions, fine needle (23G) aspiration is performed, as described by Eddyani M et al. [9.]The contents of the needle and syringe are expelled into a dry tube. After centrifugation, the supernatant is

discarded and a portion of the remaining pellet is smeared on a slide for direct examination. The remaining residue is sent to Yaounde for further tests.

In cases of ulcerative lesions or patients who have had surgery for a non-ulcerative lesion, a punch biopsy is performed using a cylindrical biopsy trocar specifically designed for this purpose, to remove a small piece of tissue about 4 mm in diameter. The biopsy will be performed in the periphery of the lesion if it is ulcerative, and 1-2 cm from the center of the lesion in the case of a non-ulcerative lesion. The sample will be fixed by immersion in 4% formaldehyde. In the case of surgical debridement, punch biopsy will be performed on the excised tissues. The biopsy will not be done in the case of a lesion in a sensitive area such as the face or inguinal area.

ZN Akonolinga:
The Ziehl-Neelson staining procedure follows that described in the WHO guidelines (Appendix 5) [3].The results are reported in a semi-quantitative manner as the number of bacilli found (1-9 / 100 fields: exact figure 10-99/100 fields: +; 1-10/fields: + +;> 10/field + + +).

Transport of samples:
Samples are transported at least once a week from the Akonolinga Center to the Centre Pasteur of Cameroon, and are maintained at a temperature between 2 and 8 ° C.

ZN and PCR examination at Centre Pasteur of Cameroon: follow standard procedures in effect at CPC.

Histopathology on punch biopsy :
After collection, samples will be kept in 4% formaldehyde until they arrive in Yaounde. The slides will be prepared by Dr. Atangana (Centre Pasteur Cameroon). Staining by hematoxylin/eosin and Ziehl-Neelson be performed for all samples. Additional staining (Giemsa or silver staining) will be performed in specific cases of clinical suspicion (leishmaniasis, mycological infection). A first reading will be done on site. The slides and the rest of the paraffin block will then be sent to the pathology department of the University Hospital of Geneva for review according to the terms of the collaboration agreement.

3.10.2 Other laboratory tests

Wound swabs for bacterial culture and mycological culture (not systematic)
Fasting plasma glucose (for diabetes screening)
Rapid Syphilis Test (differential diagnosis with yaws)
HIV testing with two rapid tests, after counseling and consent of the patient
In suspected cases of sickle cell disease and in cases of osteomyelitis: Emmel test and hemoglobin electrophoresis when Emmel test positive

3.10.3 Quality Control

Quality control for the direct examination in Akonolinga is done at the Centre Pasteur of Cameroon (PCC). Each month, 10 slides are selected randomly from among the negative exam results and 10 from among the weakly positive results to be reread at the CPC. In situations where there are fewer than 20 tests per month, all smears are reread. The CPC is also involved in external quality control conducted by the Atwerp Institute of Tropical Medicine.

3.11 Clinical opinion

In Akonolinga, the patient will be evaluated by an initial clinician, who will also fill out the data collection sheet. The initial clinician will give his/her opinion on the probability of diagnosis of Buruli (very likely, likely, possible, ruled out) before the results of additional tests are back. The same day, the patient will be reviewed by a second clinician who has not previously studied the patient's record. This other clinician will also assess whether the lesions observed correspond, in his opinion, to Buruli lesions. This second opinion will be recorded in a record kept separate from the patient record.

A summary of the case, outlining the patient's history, the first clinician's description of the wounds and two photographs of the wound, will be submitted for review to two expert dermatologists who don't have access to the results of the diagnostic tests. They will give separate opinions on the probability of diagnosis of Buruli ulcer, as well as on the differential diagnosis.

3.12 Patient Monitoring

Patients will be cared for in accordance with MSF protocol in effect in Akonolinga. Pending the results of investigations, all patients suspected of infection with M. ulcerans will be treated in the Buruli wing with appropriate wound care. If investigations reveal another diagnosis than infection with *M. ulcerans* or an underlying infection, the patient will be referred to the Akonolinga General Hospital for further treatment.

3.13 Statistical analysis.

A. **Reference standard (gold standard).**

The lack of reference standard for diagnosis of M. ulcerans infection is a major problem. The lack of reference standard is also a barrier to performance evaluation of new diagnostic tests. Latent class analysis is a statistical tool that allows this type of assessment. These models have been recently used for the diagnosis of tuberculosis in children. In this study we propose the use of a latent class model to estimate the performance of different diagnostic tests for infection with *M. ulcerans.*

B. **Concept of latent variable.**

The state of *M. ulcerans* infection is not directly observable in a patient, in which case we say that the state is latent. In this statistical approach, we postulate the existence of a latent variable representing the non-observable state of *M. ulcerans* infection (sick, not sick).

C. **Principle of the latent class model.**

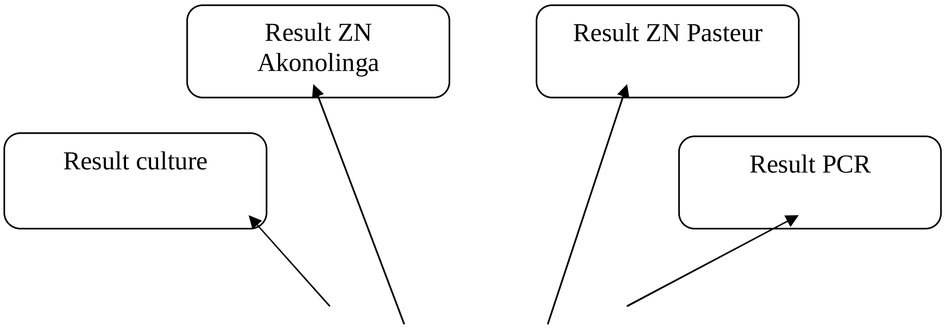

M. ulcerans infection
(Latent variable)

Figure 1. Modeling principle for a latent variable depending on the different outcomes of diagnostic tests.

The results of various diagnostic tests directly observable in a patient (Ziehl-Neelsen Akonolinga, Ziehl-Neelsen Pasteur, PCR, culture, etc.) will be grouped by "pattern" of answers to the tests (for example, "positive, positive, negative, positive " in the case of 4 tests) with the respective observed frequency.
Using the method of maximum likelihood, estimates of sensitivity and specificity of each test will be obtained. In addition, we will obtain an estimate of the a priori probability of being in the "patient" class, that is to say the prevalence of the disease. Then, for each "pattern" of answers to the tests, an a posteriori probability of being in the "patient" class will be calculated using Bayes rule, based on estimates of prevalence, sensitivity and specificity of each test.

Finally, we obtain an estimate of:

- The prevalence of infection with M. ulcerans
- The sensitivity and specificity of diagnostic tests,
- The likelihood of infection with M. ulcerans based on responses to diagnostic tests.

A strong assumption of latent class analysis is that pending the state of M. ulcerans infection, the results of the tests are independent, and knowledge of the results of one test gives no information on the results of other tests.

D. **Analysis of clinical variables and choice of final model.**

The list of clinical variables to be investigated is listed in Appendix 1. They are grouped into several categories: Patient history, general clinical examination and examination of the lesion .These variables are initially ranked according to the size of their correlation estimated by the literature and by opinions of experts on *M. ulcerans* infection (Appendix 1).

Given that the variables describing the ulcerative and non-ulcerative lesions are of different types, and that the majority of lesions are ulcerative, the model will be built initially for ulcerative lesions. A second model will be built for non-ulcerative lesions afterwards depending on the amount of information available.

Initially, only the variables whose association is recognized as intermediate or high with *M. ulcerans* will be included in the model. The contribution of tests to the model will be tested with likelihood ratio tests and

those tests that do not contribute significantly to the model will be removed. To ensure convergence of the model, it will be necessary to include in any model at least 3 diagnostic tests.

e. **Observed estimation and construction of the diagnostic score**

Once the final model has been chosen, the probability of infection with *M. ulcerans* will be calculated from the model estimates (prevalence, sensitivity and specificity of each test) for each possible combination of test responses. An algorithm will then be built based on the likelihood of infection with *M. ulcerans* and different treatment thresholds previously defined, using positive and negative likelihood ratios of different variables used in the model.

4 Authorization and Consent

The research protocol will be submitted to the National Ethics Committee of Cameroon, the Central Commission on Human Subject Research Ethics of the Geneva University Hospital, and the Ethics Committee of Médecins Sans Frontières. Permission to carry out the study will be requested from the Ministry of Health, in the framework of the National Buruli Control Program, as well as from the health authorities of the Akonolinga District and from the Akonolinga Hospital administration.

Patients (or their legal representative in case of minor patients) will be informed of the study by an information sheet they can keep. Their written consent will be sought for use of their data, performance of the punch biopsy and for sending biopsies to Switzerland.

Any future use not initially planned in the study protocol will be submitted for agreement to the National Ethics Committee of Cameroon, as well as written authorization by MSF and Epicentre. Any commercial use of the samples is forbidden.

The database will be anonymized before sending it outside of the project. Patient data is confidential and will not be disclosed to anyone outside of the study team and the MSF project. MSF-Cameroon remains the owner of the data transmitted.

5 Analysis of risks and benefits

Expected benefits for the patient:
Patients who participate in the study will benefit from an improved diagnosis of there skin lesions. Additionally, treatment of their skin lesion will be taken care of by MSF, independently from the diagnosis of *Mycobacterium ulcerans* infection.

Expected risks for the patient:
Additional risks for the patient related to his participation in the study are the risks related to the skin biopsy, namely a minimal risk of bleeding or of infection. This risk will be minimised through the appropriate training on the technique by an dermatologist experienced in this procedure.

Expected benefits for the community:
The community served by Akonolinga Hospital will benefit from a better access to care for chronic skin wounds as part of the study, independently from the diagnosis of *Mycobacterium ulcerans* infection. Besides, the study aims to establish a simplified management algorithm, which would limit the use of supplementary tests, and which could be deployed at a more peripheral level of the health care system. We

hope that this will improve early diagnosis of *Mycobacterium ulcerans* infections, which is a key element in treatment success.

The staff of the “Buruli pavillon” will benefit from ongoing training management of chronic skin lesions, done by an expatriate dermatologist. Besides, Akonolinga laboratory will benefit from a training at the Centre Pasteur Cameroon, to improve its capacity to perform diagnostic tests for Buruli. The laboratory will also benefit from a follow up of this training through ongoing quality control.

6 Financing

Funding for the materials, performance of diagnostic tests in Cameroon, the transport of samples and treatments is supported by Médecins Sans Frontières. A request to the humanitarian fund HUG is underway to cover possible additional costs of HUG pathologists: 5000 CHF for preparation of paraffin blocks and special stains. Costs can increase if the recruitment of non-Buruli cases (Buruli = differential diagnosis) increases. A request will be made to the humanitarian fund HUG for doctors in the field to participate in medical and treatment training and for field visits of experts from Geneva.

7 References

Reference List

(1) Diseases covered by NTD Department.. WHO. [2007] 5-3-2010.
Ref Type: Internet Communication

(2) Ellen DE, Stienstra Y, Teelken MA, Dijkstra PU, van der Graaf WT, van der Werf TS. Assessment of functional limitations Caused by Mycobacterium ulcerans infection: towards a Buruli ulcer functional limitation score. *Trop Med Int Health.* 2003, 8:90-96.

(3) Portaels, F., Johnson, P, and Meyers, WM Buruli ulcer, diagnosis of Mycobacterium ulcerans disease.A manual for health care providers. 2 WHO.
Ref Type: Generic

(4) Mensah-Quainoo E, Yeboah-Manu D, Aseb C et al.Diagnosis of Mycobacterium ulcerans infection (Buruli ulcer) at a Treatment Centre in Ghana: a retrospective analysis of laboratory results of Clinically Diagnosed boxes. *Trop Med Int Health.* 2008, 13:191-198.

(5) Herbinger KH, Adjei O, wua NY-Boateng et al. Comparative Study of the sensitivity of different diagnostic methods for diagnosis of Buruli The Laboratory ulcer disease. *Clin Infect Dis.* 2009, 48:1055-1064.

(6) Phillips R, Horsfield C, Kuijper S et al.Sensitivity of PCR Targeting the IS2404 insertion sequence of Mycobacterium ulcerans in Assay using punch biopsy year specimens for diagnosis of Buruli ulcer. *J Clin Microbiol.* 2005, 43:3650-3656.

(7) Eddyani M, Debacker M, Martin A et al.Primary culture of Mycobacterium ulcerans from human tissue specimens after storage in semisolid transport medium. *J Clin Microbiol.* 2008; 46:69-72.

(8) Phillips RO, Sarfo FS, Osei-Sarpong F et al. Sensitivity of PCR targeting Mycobacterium ulcerans by use of fine-needle aspirates for diagnosis of Buruli ulcer. *J Clin Microbiol.* 2009; 47:924-926.

(9) Eddyani M, Fraga AG, Schmitt F et al.Fine-needle aspiration sampling technique for efficient year bacteriological diagnosis of Buruli ulcer nonulcerative. *J Clin Microbiol.* 2009, 47:1700-1704.

(10) Bretzel G, Siegmund V, Nitschke J, et al.A Stepwise Approach To The laboratory diagnosis of Buruli ulcer disease. *Trop Med Int Health.* 2007; 12:89-96.

(11) FA Klok, Mos IC, Nijkeuter M et al.Simplification Of The revised Geneva score for Assessing clinical probability of pulmonary embolism. *Arch Intern Med.* 2008; 168:2131-2136.

(12) Porten K, Sailor K, Earl E et al.Buruli ulcer in prevalence of Akonolinga Health District, Cameroon: results of a cross sectional survey. *PLoS Negli Too Dis.* 2009; 3: E466.

(13) MSFCH. Annual report 2009 program; Buruli Akonolinga project, Cameroon. [2007]
Ref Type: Report

(14) Chappuis, F. Comte, E. Vuagnat, H. Loutan, L., and Tamrat, A. Neglected tropical diseases: a decade of partnership with Doctors Without Borders.Revue Médicale Suisse 2009 [5], S32-S34. 2009- 11-10-2010.
Ref Type: Journal (Full)

7 Appendix

Appendix 1: Variables to explore for the construction of a diagnostic score for infection with *M. ulcerans.*
Appendix 2: Information Sheet
Appendix 3. Consent Form

Appendix 1: Variables to explore for the construction of a diagnostic score of infection with *M. ulcerans.*

	**Priority**
**History:**	
History of Buruli, an old scar	1
History of cases of Buruli under the same roof	1
Knowledge of cases of Buruli ulcer in the same village	1
Duration of current episode	1
Painful lesions	1
History of fever or fever during examination	1
Itching at the lesion site	2
Prior treatment for this lesion	3
History of trauma at the site of the wound if (insect bite, injury, burn)	3
**Clinical examination on admission:**	
Location of lesions	1
Perilesional edema Yes / No	1
Type of lesion: ulcerative/ non-ulcerative	1
In cases of ulcerative lesion: the presence of fibrin	1
In cases of ulcerative lesion: necrosis wet/dry	1
In cases of ulcerative lesion: undermining - undermined edges of the ulcer	1
In cases of ulcerative lesion: underlying induration	1
If the lesion is non-ulcerative: type of lesion: nodule/ plaque/localized oedema	1
If the lesion is non-ulcerative: mobility compared to the skin and underlying tissues	1
If the lesion is non-ulcerative: tenderness: yes/no	1
If the lesion is non-ulcerative: temperature of the lesion: normal/hot	1
Vascular status: normal/abnormal	2
Locoregional lymph nodes	2
Complication and type of complication: secondary infection, malignancy, limited joint mobility	2
Lesion size (cm), according to the average of the 2 largest diameters measured at 90 ° from each other (undermining must be taken into account as well as the underlying induration that has not yet come off)	2
Bone involvement Yes/No	2
In cases of ulcerative lesions: base of ulcer white/red	2
If the lesion is non-ulcerative color: depigmented/ hyperpigmented/erythema	2
If the lesion is non-ulcerative: well demarcated/ defined: yes/no	2
Signs of peripheral neuropathy (distal hyposensitivity)	3
Depth of lesion (skin (stage 1), dermis (2), hypodermis (3), fascia, muscle, (4))	3
In cases of ulcerative lesion: exudate<br>quantity: none, low, high<br>Quality: serous, bloody, purulent	3

In cases of ulcerative lesion: odor	3
In cases of ulcerative lesion: bleeding	3
In cases of ulcerative lesion: periphery inflammatory Yes/No	3
If the lesion is non-ulcerative: consistency: indurated/padded/soft	3

Reference List

(1) Canavate C, Herrero M, Nieto J, Cruz I, Chicharro C, Aparicio P, et al. Evaluation of two rK39 dipstick tests, direct agglutination test, and indirect fluorescent antibody test for diagnosis of visceral leishmaniasis in a new epidemic site in highland Ethiopia. Am J Trop Med Hyg 2011 Jan;84(1):102-6.

(2) Tuyisenge L, Ndimubanzi CP, Ndayisaba G, Muganga N, Menten J, Boelaert M, et al. Evaluation of latent class analysis and decision thresholds to guide the diagnosis of pediatric tuberculosis in a Rwandan reference hospital. Pediatr Infect Dis J 2010 Feb;29(2):e11-e18.

Formatted: Left

Formatted: Space After: 12 pt

Deleted: Reference List¶
¶
(1) Canavate C, Herrero M, Nieto J, Cruz I, Chicharro C, Aparicio P, et al. Evaluation of two rK39 dipstick tests, direct agglutination test, and indirect fluorescent antibody test for diagnosis of visceral leishmaniasis in a new epidemic site in highland Ethiopia. Am J Trop Med Hyg 2011 Jan;84(1):102-6.¶
(2) Tuyisenge L, Ndimubanzi CP, Ndayisaba G, Muganga N, Menten J, Boelaert M, et al. Evaluation of latent class analysis and decision thresholds to guide the diagnosis of pediatric tuberculosis in a Rwandan reference hospital. Pediatr Infect Dis J 2010 Feb;29(2):e11-e18.¶
¶
